# The Predictive Value of the NICE “Red Traffic Lights” in Acutely Ill Children

**DOI:** 10.1371/journal.pone.0090847

**Published:** 2014-03-14

**Authors:** Evelien Kerkhof, Monica Lakhanpaul, Samiran Ray, Jan Y. Verbakel, Ann Van den Bruel, Matthew Thompson, Marjolein Y. Berger, Henriette A. Moll, Rianne Oostenbrink, for the European Research Network on recognising serious InfEctions (ERNIE) members

**Affiliations:** 1 Erasmus MC-Sophia Children's Hospital, Department of General Pediatrics, Rotterdam, The Netherlands; 2 Department of General and Adolescent Pediatrics, University College London, Institute of Child Health, London, United Kingdom; 3 Pediatric Intensive Care Unit, Great Ormond Street Hospital, London, United Kingdom; 4 Department of General Practice, Katholieke Universiteit Leuven, Leuven, Belgium; 5 Department of Primary Care Health Sciences, University of Oxford, Radcliffe Observatory Quarter, Oxford, United Kingdom; 6 Department of General Practice, University Groningen, University Medical Centre Groningen, Groningen, The Netherlands; University of British Columbia, Canada

## Abstract

**Objective:**

Early recognition and treatment of febrile children with serious infections (SI) improves prognosis, however, early detection can be difficult. We aimed to validate the predictive rule-in value of the National Institute for Health and Clinical Excellence (NICE) most severe alarming signs or symptoms to identify SI in children.

**Design, Setting and Participants:**

The 16 most severe (“red”) features of the NICE traffic light system were validated in seven different primary care and emergency department settings, including 6,260 children presenting with acute illness.

**Main Outcome Measures:**

We focussed on the individual predictive value of single red features for SI and their combinations. Results were presented as positive likelihood ratios, sensitivities and specificities. We categorised “general” and “disease-specific” red features. Changes in pre-test probability versus post-test probability for SI were visualised in Fagan nomograms.

**Results:**

Almost all red features had rule-in value for SI, but only four individual red features substantially raised the probability of SI in more than one dataset: “does not wake/stay awake”, “reduced skin turgor”, “non-blanching rash”, and “focal neurological signs”. The presence of ≥3 red features improved prediction of SI but still lacked strong rule-in value as likelihood ratios were below 5.

**Conclusions:**

The rule-in value of the most severe alarming signs or symptoms of the NICE traffic light system for identifying children with SI was limited, even when multiple red features were present. Our study highlights the importance of assessing the predictive value of alarming signs in clinical guidelines prior to widespread implementation in routine practice.

## Introduction

Fever is one of the most common symptoms among children presenting to ambulatory care.[1]–[3] The majority of children presenting with an acute illness to ambulatory care will have self-limiting viral infections, with only a small proportion having a serious infection (SI).[1], [4]–[6] Early recognition and treatment of children with SI are related to better prognosis,[7], [8] however identification of SI at first presentation can be difficult.

The National Institute for Health and Clinical Excellence (NICE) 2013 guideline for the management of children with feverish illness provides comprehensive guidance on the assessment, investigation and management of children presenting at different settings, including primary care and pediatric specialty settings.[6], [9] One of the key elements of the guideline is a “traffic light” system for the diagnostic assessment of children under five years of age presenting with a feverish illness. This evidence and consensus-based system includes clinical features identified from existing scoring systems for acutely ill children,[10]–[13] and disease-specific signs and symptoms. Children with the most alarming (or “red”) features are considered at higher risk of SI, for whom subsequent management includes invasive investigations, treatment, and hospital admission.

As one of the few evidence-based guidelines for children with fever [14], [15] and the only for both primary and secondary care, the NICE febrile child guideline has been implemented in many settings in not only the United Kingdom but also in other countries. Recently, two studies reported low specificities for the approach that any abnormal amber or red feature would indicate possible SI.[16], [17] This could be due to the inclusion of amber features, whose association with SI may be weaker.

In this study we aimed to determine the predictive ("rule-in") value of the red features of the NICE traffic light system, both for the individual red features as their combinations for identifying children with SI in various acute pediatric settings in Europe.

## Methods

### Identification of datasets

We used data on seven independent cohorts [4], [18]–[23] collected by collaborators of the European Research Network on recognising serious InfEctions (ERNIE) group.[24] Data were prospectively collected at first contact using standardised (site-specific) documentation of patient characteristics, except for Monteny et al [19] where data was collected using structured clinical proformas separate from the consultation. All datasets were cohort studies of children in various age ranges (0–16 years), presenting to ambulatory care settings (i.e. general or family practice, pediatric outpatient clinic, pediatric assessment unit or emergency department) with an acute illness or infection.

Two datasets based on primary care settings were considered as low prevalence settings of SI (<5%) and five datasets based on emergency care settings as high prevalence settings (>5%).[25] More details on the original cohorts have been published elsewhere ([4], [18]–[23]).

### Ethical approval

This research conforms to the Helsinki Declaration and to local legislation. The original study authors have all agreed to share their data, and had obtained ethical approval from their local research ethics committees for the initial data collection, prior to this study.

### Processing of included datasets

Key characteristics of each dataset are shown in table 1. We selected children under the age of five years with an acute illness based on general symptoms [4], [21], [22] or specifically on the presence of fever [18]–[20], [23], as this is the target group of the NICE guideline (table 1).

**Table 1:** Characteristics of datasets with children <5 years of age suspected of acute illness*.

Dataset	Setting	Country	Inclusion Criteria	Exclusion Criteria	n/ original study population (%)	Prevalence Serious Infection % (CI)	Median Age (IQR)
1. Bleeker et al. 2007[23]	ED	NL	Children aged 1–36 months with fever (T>38) at emergency department, no clear focus identified after evaluation GP or history by pediatrician	Chronic disease, Immunodeficiency	595/595 (100)	23.0 (19.6–26.4)	0.8 (0.3–1.4)
2. Brent et al. 2011[18]	ED	UK	All children presenting with a medical problem to the pediatric emergency-care unit whatever their age	Children who required immediate resuscitation. Comorbidity and chronic illness	494/2777 (18)	9.3 (6.7–11.9)	1.5 (0.9–2.7)
3. Oostenbrink et al. 2004[22]	ED	NL	Children aged 1 month-16 years, meningeal signs at GP, pediatrician or self-referred with neck pain	Comorbidity, ventriculoperitoneal drain	423/593 (71)	40.2 (35.5–44.9)	1.1 (0.5–2.7)
4. Roukema et al. 2008[20]	ED	NL	Children aged 1 month-16 years with fever (T>38) at emergency department, without meningeal irritation	Chronic disease, Immunodeficiency	1459/1750 (83)	11.7 (10.1–13.4)	1.4 (0.7–2.6)
5. Thompson et al. 2009[21]	PAU	UK	Children aged 3 months-16 years with suspected acute infection	Children with diseases liable to cause repeated serious bacterial infection, and infections resulting from penetrating trauma	434/700 (62)	37.1 (32.5-41.7)	1.6 (0.9–2.7)
6. Monteny et al. 2008[19]	GP	NL	Children aged 3 months-6 years, contacting a GP cooperative after hours with fever as the presenting symptom	Language barriers, no repeated inclusion within the last two weeks	487/506 (96)	4.1 (2.3-5.9)	1.7 (0.9–3.0)
7. Van den Bruel et al. 2007[4]	GP/AP/ ED	BE	Non-referred children ≤16 years with acute illness for max 5 days	Traumatic or neurological illness, intoxication, psychiatric or behavioural problems without somatic cause or an exacerbation of a chronic condition. No repeated inclusion of same infant within 5 days. Exclusion of physicians if the assumption of consecutive inclusion was probably violated	2368/3891 (60)	1.2 (0.8–1.6)	1.9 (0.9–3.2)

*Using temperature >38.0°C or general symptoms of acute illness.

GP  =  General Practice; AP  =  Ambulatory Pediatric Care; ED  =  Emergency Department; PAU  =  Pediatric Assessment Unit; BE  =  Belgium; NL  =  the Netherlands; UK  =  United Kingdom; CI  =  Confidence Interval IQR =  interquartile range.

The NICE traffic light system includes 16 red features, which are categorised into 5 main domains: Colour (1 red feature), Activity (4 red features), Respiratory (3 red features), Hydration (1 red feature), and Other (7 red features).[6],[9] When study variables were not entirely identical to the red features in the NICE febrile child guideline, we identified proxies where possible. Identification and handling of variables has been described earlier [17], a full list of all approximations is described in table S1. When a red feature was not recorded in the dataset and no suitable proxy was identified, this item was excluded from that specific dataset. Table S2 outlines the unrecorded and missing data from each dataset separately.

Missing values were not imputed because the necessary missing-at-random assumption was likely to be incorrect. We considered red features that were “not documented” in individual patient records as “absent”, given that the red feature or its proxy was recorded in that particular dataset.[17]

The translation, recoding and data-checking were performed by two authors (EK, JV) and the results of each step were discussed with all primary study authors.[17]

### Outcome measures

Serious infections (SI) were defined as sepsis (including bacteremia), meningitis, pneumonia, osteomyelitis, cellulitis, and complicated urinary tract infections. [25] Serious infections (SI) were not only based on clinical diagnosis, but reference standard test criteria were used to determine final diagnoses of SI. Detailed description on these reference standard test criteria are available in the original study papers.[4], [18]–[23] Assessment of the diagnoses to ensure comparability of outcomes was discussed with the lead investigator of each study as described earlier.[17]

### Statistical analysis

The individual red features were analysed in every dataset separately. Additionally, results were categorised as “general” red features (items 1–7 and 9–10) and “disease-specific” red features (items 8 and 11–16).

We assessed the rule-in value for SI for each red feature separately by calculating positive likelihood ratios (LR+). Red features were considered to have rule-in value if they raised the probability of illness with a positive likelihood ratio of more than 5.0.[25] The univariable association between each individual red feature and the presence of SI was tested by Chi-square analysis. Likelihood ratios, sensitivity and specificity were measured for the presence of ≥1 RTL, ≥2 RTLs and ≥3 RTLs. The sensitivity and specificity for “general” and “disease-specific” red features were plotted in receiver operating characteristic (ROC) space.

The incremental diagnostic value for up to more than four red features compared to one red feature was evaluated by logistic regression analyses with forward selection (Wald test, p-value <0.05).

We visualised the change in pre-test probability versus post-test probability for SI in a Fagan nomogram.[26]

No overall pooled likelihood ratios were calculated because of the substantial clinical heterogeneity between datasets (differences in setting, inclusion criteria, immunisation schedules and definition of serious infection).[17] All analyses were done with SPSS software (version 20.0, SPSS Inc, Chicago).

## Results

### Included datasets

We selected 6,260 children under five years of age of seven pre-existing datasets (n = 6,260/10,812, 58%) for diagnostic studies in children with an acute illness (table 1). Children were included based on fever,[19], [20], [23] acute illness,[4], [18] acute infection,[21] and referral for meningeal signs.[22] Children with various severities of co-morbidity were excluded in five studies,[4], [19]–[23], one study excluded children if the acute episode was caused by an exacerbation of a chronic condition[4] and one study excluded children who required immediate resuscitation [18] (table 1). All studies included sepsis, meningitis, pneumonia and complicated urinary tract infections in their outcome definition. Osteomyelitis and cellulitis were explicitly mentioned in five and three datasets, respectively.

The median age of the selected children ranged from 0.8 years to 1.9 years. The prevalence of SI ranged from 1.2% to 4.1% in two datasets from general practice [4], [19] and from 9.3% to 40.2% in five datasets from emergency departments and a pediatric assessment unit [18], [20]–[23].

### Red traffic lights included in the datasets

Data on all red features included in domains “Colour” and “Hydration” were available in all datasets. The red features “no response to social cues”, and “weak, high-pitched or continuous cry” of domain “Activity” were not recorded in two [20], [23], and one dataset [18], respectively. Other red features in this domain were available in all datasets. Red features related to the “Respiratory” domain were not recorded in four (“grunting”) [4], [21]–[23], one (“tachypnoea”) [22], and two (“chest indrawing”) [22], [23], datasets respectively. “Disease-specific” red features (items 8 and 11–16) were recorded less frequently in all datasets but in particular in low prevalence settings (range missing values 0–50%), see table S2).

### Performance of individual red traffic lights

Table 2 shows positive and negative likelihood ratios of the 16 individual red features for each dataset separately. All red features with high rule-in value (LR+ >5) are highlighted in bold.

**Table 2:** Likelihood ratios individual red traffic lights.

positive likelihood ratio (LR+) and negative likelihood ratio (LR-)																			
Dataset	LR (CI)	prevalence																	
			COLOUR	ACTIVITY				RESPIRATORY			HYDRATION	OTHER							
			Pale/ mottled/ ashen/ blue	No response to social cues	Ill appearance	Does not (stay) wake	Weak/ high/ continuous cry	Grunting	Tachypnoea	Chest indrawings	Reduced skin turgor	Age <3 m & temp ≥38°C		Non-blanching rash	Bulging fontanelle	Neck stiffness	Status epilepticus	Focal neurologic signs	Focal seizures
Bleeker et al.[23]	LR +	High	2.3 (1.5–3.4)*	-	1.4 (1.2–1.7)*	2.6 (1.0–6.9)*	1.1 (0.9–1.3)	-	3.0 (1.9–4.6)*	-	1.9 (1.3–2.8)*	1.5 (1.1–2.1)*		-	1.8 (0.7–4.4)	-	-	-	-
(n = 595)	LR -		0.9 (0.8–0.9)	-	0.7 (0.6–0.9)	1.0 (0.9–1.0)	0.9 (0.8–1.1)		0.8 (0.8–0.9)		0.9 (0.8–1.0)	0.9 (0.8–1.0)		-	1.0 (0.9–1.0)	-	-	-	-
Brent et al.[18]	LR +	High	2.6 (1.8–3.8)*	1.6 (0.4–7.0)	2.7 (2.0–3.6)*	1.4 (0.2–11.1)	-	**7.8 (2.2–28.0)** *	2.9 (1.7–4.9)*	4.9 (2.2–10.8)*	**9.7(0.6–153.1)** *	1.9 (0.6–6.5)		**	**	**	**	**	**
(n = 494)	LR -		0.7 (0.5–0.9)	1.0 (0.9–1.0)	0.5 (0.4–0.7)	1.0 (1.0–1.0)	-	0.9 (0.8–1.0)	0.8 (0.6–0.9)	0.9 (0.7–1.0)	1.0 (0.9–1.0)	1.0 (0.9–1.0)		**	**	**	**	**	**
Oostenbrink et al. [22]	LR +	High	1.9 (1.0–3.5)*	1.9 (1.4–2.5)*	3.0 (0.8–11.7)	**7.8 (4.4–13.6)** *	1.1 (0.7–1.8)	-	-	-	**7.4 (1.7–33.5)** *	1.6 (0.9–2.9)		**7.2 (3.3–15.9)** *	2.7 (1.6–4.4)*	1.8 (1.6–2.0)*	-	**7.4 (2.6–21.4)** *	**
(n = 423)	LR -		0.9 (0.9–1.0)	0.8 (0.7–0.9)	1.0 (0.9–1.0)	0.6 (0.6–0.7)	1.0 (0.9–1.1)	-	-	-	0.9 (0.9–1.0)	0.9 (0.9–1.0)		0.8 (0.8–0.9)	0.8 (0.8–0.9)	0.2 (0.1–0.3)	-	0.9 (0.8–0.9)	**
Roukema et al. [20]	LR +	High	1.5 (0.2–12.8)	-	2.7 (1.6–4.5)*	2.8 (1.2–6.5)*	2.9 (1.4–6.3)*	2.9 (1.6–5.3)*	2.6 (1.4–4.7)*	1.2 (0.7–2.1)	1.9 (0.2–16.8)	1.8 (1.0–3.3)*		**7.5 (1.5–37.0)** *	**11.3(1.9–67.1)** *	4.8 (1.9–12.2)*	**	**5.0 (2.5–10.2)** *	1.5 (0.2–12.8)
(n = 1459)	LR -		1.0 (1.0–1.0)	-	0.9 (0.9–1.0)	1.0 (1.0–1.0)	1.0 (0.9–1.0)	0.9 (0.9–1.0)	1.0 (0.9–1.0)	1.0 (0.9–1.0)	1.0 (1.0–1.0)	1.0 (0.9–1.0)		1.0 (1.0–1.0)	1.0 (1.0–1.0)	1.0 (1.0–1.0)	**	0.9 (0.9–1.0)	1.0 (1.0–1.0)
Thompson et al.[21]	LR +	High	1.3 (1.0–1.6)	2.4 (0.9–6.2)	**	1.5 (0.9–2.5)	1.5 (1.1–2.1)*	-	4.7 (1.5–14.4)*	1.5 (1.0–2.3)	2.0 (0.8–4.9)	**		1.7 (0.9–3.4)	-	**	-	-	-
(n = 434)	LR -		0.9 (0.8–1.0)	1.0 (0.9–1.0)	**	0.9 (0.9–1.0)	0.9 (0.8–1.0)	-	0.9 (0.9–1.0)	0.9 (0.8–1.0)	1.0 (0.9–1.0)	**		1.0 (0.9–1.0)	-	**	-	-	-
Monteny et al.[19]	LR +	Low	1.7 (1.1–2.5)*	1.4 (0.9–2.1)	1.7 (0.6–4.9)	1.8 (1.0–3.2)	0.8 (0.5–1.4)	1.5 (1.2–1.8)*	**	**	0.8 (0.1–5.4)	**		0.4 (0.1–2.8)	**	1.8 (1.1–2.9)*	-	-	-
(n = 487)	LR -		0.7 (0.4–1.1)	0.7 (0.5–1.2)	0.9 (0.8–1.1)	0.8 (0.5–1.1)	1.2 (0.8–1.7)	0.4 (0.1–1.0)	**	**	1.0 (0.9–1.1)	**		1.1 (1.0–1.2)	**	0.7 (0.4–1.1)	-	-	-
Van den Bruel et al.[4]	LR +	Low	**83.6 (12.2–572.4)** *	3.2 (1.9–5.3)*	**6.6 (4.5–10.0)** *	**5.9 (3.5–10.0)** *	**7.0 (4.1–11.8)** *	-	**7.3 (4.1–12.9)** *	**8.0 (4.9–13.1)** *	2.2 (0.3–15.5)	**9.3 (2.3–38.1)** *		**	-	**	-	-	-
(n = 2368)	LR -		0.9 (0.8–1.0)	0.7 (0.5–1.0)	0.5 (0.4–0.8)	0.7 (0.5–0.9)	0.7 (0.5–0.9)	-	0.7 (0.6–0.9)	0.6 (0.5–0.9)	1.0 (0.9–1.1)	0.9 (0.8–1.0)		**	-	**	-	-	-

‘General’ red traffic lights: Pale/ mottled/ ashen/ blue; No response to social cues; Ill appearance; Does not (stay) wake; Weak/ high/ continuous cry; Grunting; Tachypnoea; Reduced skin turgor; Age <3 m & temp ≥38°C.

‘Disease specific’ red traffic lights: Chest indrawings; Non-blanching rash; Bulging fontanelle; Neck stiffness; Status epilepticus; Focal neurologic signs; Focal seizures.

CI: confidence interval.

*P-value <0.05 (Chi-square analysis).

**Red traffic light not positive in non SI- and/or SI population.

-Not recorded.

Four of all 16 red features did not achieve high rule-in value (LR+ <5) including two red features which were not available in the datasets or were not reaching significance (p<0.05) when present.

The one red feature which provided high rule-in value in two datasets from both low and higher prevalence settings, was “does not wake or if roused does not stay awake” (LR+5.9 (95% CI 3.5–10.0) and LR+7.8, 95% CI 4.4–13.6, respectively). The red features “reduced skin turgor”, “non-blanching rash”, and “focal neurological signs” showed high rule-in value in two high prevalence settings each (range LR+5.0-9.7)[18], [20], [22]. The red features “pale/mottled/ashen/blue”, “appears ill to a healthcare professional”, “weak, high-pitched or continuous cry”, “tachypnoea”, “moderate or severe chest indrawing”, and “age 0–3 months & temperature ≥38°C” showed high rule-in value in one low prevalence setting (range LR+5.9-83.6)[4]. High rule-in value for the red features “grunting” and “bulging fontanelle”, was observed in one high prevalence dataset (range LR+7.8–11.3).[20] In two high prevalence settings for none of the red features high rule-in value was observed.[21], [23]

### Performance of multiple red traffic lights

**Figure 1:**
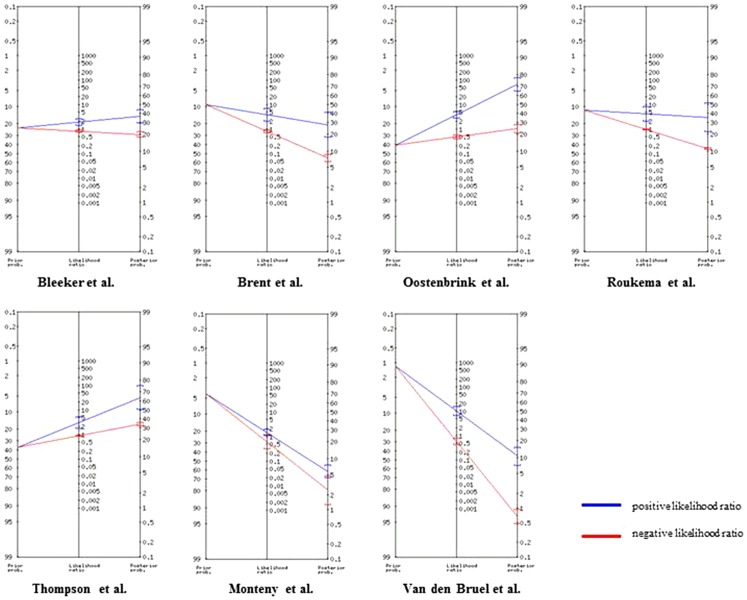
Calculation of post-test pobability for serious infections if ≥3 red traffic lights present using Fagan nomogram.

The association between SI and the number of positive red features with the performance measures of positive likelihood ratios, sensitivity and specificity is shown in table 3. We measured the maximum predictive value of multiple red features by logistic regression analysis and the slope of the ROC-curve. We noted a significant increase of rule-in value with the number of positive red features in most datasets (range LR+2.1 – 10.0 when ≥3 red features), with the exception of Monteny et al.[19] (p-value <0.05). This was also observed in the increased values of specificity when more red features were present. The presence of 4 or more red features did not contribute to discriminative value compared to up to 3 red features. The proportion of children having ≥3 red features ranged from 2% to 50% and did not differ between low and high prevalence settings. “General” red features were almost entirely responsible for the total ROC-area (table 3). We did not test disease-specific red features on disease-specific outcome measures due to the small numbers of these events. In figure 1 we visualised the change in pre-test to post-test probability for SI when three or more (general or disease-specific) red features were present in a Fagan nomogram.[27] For example, the 9% pre-test probability of having a SI for a child in the Brent et al dataset increases to 28% (95% CI 17–42%) post-test probability when having three or more red features, but decreases only to 7% (95% CI 6–9%) if less than three red features were present.

**Table 3:** Likelihood ratios and ROC-areas of combinations of multiple red traffic lights.

		BLEEKER et al.[23]	BRENT et al.[18]	OOSTENBRINK et al.[22]	ROUKEMA et al.[20]	THOMPSON et al.[21]	MONTENY et al.[19]	VAN DEN BRUEL et al.[4]
		N_total_ = 595 SI = 137;23.0%	N_total_ = 494 SI = 46;9.3%	N_total_ = 423 SI = 170;40.2%	N_total_ = 1,459 SI = 171;11.7%	N_total_ = 434 SI = 161;37.1%	N_total_ = 487 SI = 20;4.1%	N_total_ = 2,368 SI = 28;1.2%
Number red features	LR +/- (CI)							
≥1 RTL	Likelihood ratio +	1.18 (1.10–1.27)*	1.94 (1.57–2.39)*	1.39 (1.27–1.51)*	1.75 (1.39–2.20)*	1.28 (1.12–1.46)*	1.08 (1.05–1.12)	3.33 (2.76–4.02)*
	Likelihood ratio -	0.41 (0.24–0.70)	0.42 (0.26–0.69)	0.13 (0.06–0.28)	0.81 (0.72–0.91)	0.59 (0.43–0.80)	-	0.24 (0.12–0.53)
≥2 RTLs	Likelihood ratio +	1.49 (1.25–1.79)*	3.70 (2.68–5.12)*	2.36 (1.94–2.87)*	3.40 (2.24–5.16)*	1.75 (1.31–2.33)*	1.25 (1.07–1.46)	6.38 (4.82–8.44)*
	Likelihood ratio -	0.68 (0.55–0.84)	0.49 (0.35–0.69)	0.35 (0.26–0.46)	0.88 (0.82–0.94)	0.78 (0.67–0.89)	0.36 (0.10–1.35)	0.36 (0.21–0.62)
≥3 RTLs	Likelihood ratio +	2.10 (1.50–2.93)*	3.90 (2.15–7.08)	4.65 (3.29–6.58)*	4.14 (2.02–8.50)	2.97 (1.66–5.31)*	1.44 (1.07–1.95)	10.0 (6.43–15.45)*
	Likelihood ratio -	0.81 (0.72–0.91)	0.79 (0.67–0.94)	0.47 (0.39–0.57)	0.95 (0.91–1.00)	0.88 (0.81–0.95)	0.58 (0.30–1.15)	0.56 (0.40–0.79)
	Sens/Spec (CI)							
≥1 RTL	Sensitivity	0.91 (0.84-0.94)	0.74 (0.60-0.84)	0.96 (0.92-0.98)	0.36 (0.29-0.43)	0.76 (0.69-0.82)	1.00 (0.84-1.00)	0.82 (0.64-0.92)
	Specificity	0.23 (0.20-0.27)	0.62 (0.57-0.66)	0.31 (0.25-0.37)	0.80 (0.77-0.82)	0.40 (0.35-0.46)	0.07 (0.05-0.10)	0.75 (0.74-0.77)
≥2 RTLs	Sensitivity	0.58 (0.50-0.66)	0.59 (0.44-0.72)	0.76 (0.70-0.82)	0.16 (0.12-0.23)	0.40 (0.33-0.48)	0.90 (0.70-0.97)	0.68 (0.49-0.82)
	Specificity	0.61 (0.56-0.65)	0.84 (0.81-0.87)	0.68 (0.62-0.73)	0.95 (0.94-0.96)	0.77 (0.72-0.82)	0.28 (0.24-0.32)	0.89 (0.88-0.91)
≥3 RTLs	Sensitivity	0.31 (0.24-0.39)	0.26 (0.16-0.40)	0.59 (0.51-0.66)	0.06 (0.03-0.11)	0.17 (0.12-0.24)	0.70 (0.48-0.85)	0.46 (0.30-0.64)
	Specificity	0.85 (0.82-0.88)	0.93 (0.91-0.95)	0.87 (0.83-0.91)	0.98 (0.98-0.99)	0.94 (0.91-0.96)	0.51 (0.47-0.56)	0.95 (0.94-0.96)
	ROC-area (CI)							
All red features		0.64 (0.59–0.70)	0.73 (0.65–0.82)	0.79 (0.74–0.84)	0.59 (0.54–0.63)	0.63 (0.57–0.68)	0.65 (0.53–0.77)	0.84 (0.75–0.93)
General#		0.64 (0.59–0.70)	0.73 (0.65–0.81)	0.69 (0.64–0.75)	0.59 (0.54–0.64)	0.60 (0.55–0.66)	0.65 (0.54–0.76)	0.82 (0.72–0.91)
Disease spec.∧		0.51 (0.46–0.57)	0.56 (0.47–0.66)	0.77 (0.72–0.81)	0.53 (0.48–0.58)	0.56 (0.50–0.62)	0.56 (0.43–0.69)	0.69 (0.57–0.81)
								

*P-value <0.05 (added value of e.g. ≥3 RTLs above ≥2 RTLs, by logistic regression analysis).

LR: likelihood ratio (positive and negative).

Sens: sensitiviteit; Spec: specificiteit; CI: confidence interval.

# 1. Colour: pale / mottled/ ashen/ blue; 2. No response to social cues; 3. Ill appearance; 4. Does not (stay) awake; 5. Weak/ high pitched/ continuous cry; 6. Grunting; 7. Tachypnoea; 9. Reduced skin turgor; 10. Age <3 m & temp ≥38°C;

∧ 8. Chest indrawing 11. Non-blanching rash; 12. Bulging fontanelle; 13. Neck stiffness; 14. Status epilepticus; 15. Focal neurologic symptoms; 16. Focal seizures.

## Discussion

### Main findings

This is the first study on broadly validating the diagnostic performance of the individual red features and their combinations of the NICE febrile child guideline in acutely ill children in various settings in Europe. Although we observed rule-in value for almost all individual red features in at least one dataset, only four red features raised the probability of SI with a positive likelihood ratio of more than 5.0 in more than one setting: “does not wake or if roused does not stay awake”, “reduced skin turgor”, “non-blanching rash”, and “focal neurological signs”. Children with more than one red feature had an increased risk of SI, however, more than three red features did not further increase disease probability.

### Comparison with other studies

To our knowledge there are three previous studies that estimated the predictive value of any amber or red feature for the detection of SI, but they did not evaluate the individual features of the NICE traffic light system separately. De et al.[16] found that the NICE traffic light system failed to identify a substantial proportion of children with serious bacterial infections. Combining the amber and red feature categories resulted in a sensitivity of 85.8% and specificity of 28.5% for the detection of any serious bacterial infections. Within the original data of Thompson et al. the diagnostic value of vital signs and the NICE traffic light system for identifying children with SI was assessed in a pediatric assessment unit.[21] They stated that the presence of one or more amber and red features was 85% sensitive, but only 29% specific in identifying serious or intermediate infections.[21] However, this original study was performed in children up to 16 years of age in contrast to this present study limited to children up to 5 years of age. Finally, a previous study assessing the diagnostic value of any abnormal amber or red feature (not considering combinations) of the NICE traffic light system to rule-out SI, had sensitivity of 97–100% in low and intermediate prevalence settings and 87–99% in high prevalence settings.[17] The results of all three validation studies suggest possible clinical value for ruling-out SI using both amber and red features, but at the expense of a large group of children testing false positive. However, up to 15% of children with a serious infection will be missed. Alternatively, the presence of any amber or red feature does not allow ruling-in SI considering the very low specificity. In low prevalence settings, alarming signs are preferably highly sensitive to correctly rule-out SI in order to limit incorrect referral.[24] In high prevalence settings specificity is more important because a high rate of false positive children could result in high admission rates and unnecessary investigations.[24] Unfortunately there was too much heterogeneity in our datasets to stratify according to prevalence.

### Clinical and research implications

With decreasing incidence of SI, clinicians may increasingly rely on alarming symptoms described in (inter)national clinical guidelines. Broad validation could support the wider adoption of the NICE guideline in various settings in Europe and other high-income countries. Although the traffic light system of the NICE febrile child guideline is mostly based on systematic literature reviews and consensus, only four red features achieved high rule-in value in more than one dataset and none of them across all settings. Moreover, in at least as many datasets these four red features did not achieved high rule-in value and therefore hampers strong conclusions.

The rule-in value of several other red features was not confirmed in multiple settings either, questioning their inclusion in this setting-independent traffic light system.

Our observations of varying rule-in values of red features in the 7 databases did not support the development of one prediction model including the most important red features. However, we consistently observed an association between 3 or more red features and SI but combinations of red features will never be able to definitely rule-in a SI without uncertainty. This could be due to dilution of their accuracy by the inclusion of aspecific red features or because of the interaction between different red features.

The relatively lower recording of “disease-specific” features hampered our analyses, in particular in low prevalence settings. This may in part have been caused by the fact that it is more difficult to identify proxies for such features, in contrast to more general features.

The main findings in our study corresponds with the limited performance of the Yale Observation Scale, on which the NICE traffic light system is partly based.[17], [25] In the revised 2013 guideline[9] two red features were deleted of the previous 2007 protocol^6^ or transferred to amber features: “Age 3–6m & temperature ≥39°C” and “bile-stained vomiting”. This is supported by our findings that we did not find rule-in value for the former but only had one dataset available for the latter which showed high rule-in value though. Next, as disease specific red features are strongly related to specific but rare diseases, their positive documentation rate is already expected to be low. Although these disease specific red features may be relevant for one specific outcome, it is difficult to evaluate these in the general population of fever with a broad differential diagnosis. However, achieving complete certainty with clinical features is not the goal here. Rather, red features should lift the probability of SI over a certain decision threshold: either to refer, request additional testing or start empiric treatment. As we do not know at what specific risk thresholds we (intuitively) undertake action, clinical interpretation of post-test probabilities as expressed in Fagan nomograms (figure 1) remains difficult. As diagnosis assessment is a dynamic process and may be influenced by evolution of symptoms in time, repeated assessment of deviating red features in those with only one or two features in particular, may improve the evaluation of SI.

Finally, the NICE traffic light system could also be improved by taking more recent evidence into account, such as on peripheral circulation, parental concern [25] or urine analysis [16].

### Strengths and limitations

We assessed the NICE red traffic lights in 6,260 children from seven existing datasets with various pediatric populations and settings including two low prevalence primary care settings, which are usually underrepresented in diagnostic studies in this area.[24] In addition, we validated the red features separately to identify their individual predictive value.

Despite the large amount of data, not all red features had been recorded in all datasets, necessitating the use of proxy variables.[17] Furthermore, differences in population characteristics (table 1), such as age distribution or prevalence of specific diagnoses within the group of SI, prevented the calculation of overall diagnostic performance measures.

Furthermore, by assuming missing red features as not present and more complete documentation of red features in ill children, we may have overestimated our likelihood ratios by increasing the contrast between children with and without SI.

However, the variability in variables and case-mix reflects clinical practice and therefore will strengthen generalizability of our results.

### Conclusion

Our results support rule-in value of several individual red features from the NICE febrile child guideline in specific settings, although not consistent. However most features had little rule-in value across multiple settings. The NICE red traffic lights, even when three or more features are present, seem to have limited value for ruling-in serious infections. Our results underline the importance to widely validate the predictive value of individual and combinations of multiple red features in clinical guidelines, prior to widespread dissemination and adoption.

## Supporting Information

Table S1 **Variables and proxies.**
(DOC)

Table S2 **Missing/not recorded data.**
(DOC)
