# Supplementary material for: The Predictive Value of the NICE “Red Traffic Lights” in Acutely Ill Children
Source: PLoS One. 2014 Mar 14;9(3):e90847. doi: 10.1371/journal.pone.0090847 (PMC3954615; doi:10.1371/journal.pone.0090847)
Supplement: Table S1 — Variables and proxies. (DOC) [file pone.0090847.s001.doc]

**Table S1: variables and proxies**

+ Red traffic light recorded in database

- Red traffic light not recorded in database

| Dataset Variables used (original or proxy) | | | | | | | | | | | | | | | | |
| --- | --- | --- | --- | --- | --- | --- | --- | --- | --- | --- | --- | --- | --- | --- | --- | --- |
| COLOUR ACTIVITY RESPIRATORY HYDRATION OTHER | | | | | | | | | | | | | | | | |
|  | Colour | No response to social cues | Ill appearance | Does not (stay) awake | Quality of cry | Grunting | Tachypnoea | Chest indrawings | Reduced skin turgor | Age <3m & temp ≥38°C | Non-blanching rash | Bulging fontanelle | Neck stiffness | Status epilepticus | Focal neurologic signs | Focal seizures |
| Bleeker et al.^[23]^ | + | - | + | +^*^ | + | - | +^#^ | - | += | + | - | + | - | - | - | - |
|  |  |  |  |  |  |  |  |  |  |  |  |  |  |  |  |  |
| Brent et al.^[18]^ | + | + | + | + | - | + | + | + | += | + | + | + | + | + | + | + |
|  |  |  |  |  |  |  |  |  |  |  |  |  |  |  |  |  |
| Oostenbrink et al.^[22]^ | + | +^ | + | +^*^ | + | - | - | - | += | + | + | + | + | - | + | + |
|  |  |  |  |  |  |  |  |  |  |  |  |  |  |  |  |  |
| Roukema et al.^[20]^ | +^&^ | - | + | +^*^ | + | + | + | + | + | + | + | + | + | + | + | + |
|  |  |  |  |  |  |  |  |  |  |  |  |  |  |  |  |  |
| Thompson et al.^[21]^ | + | + | + | + | + | - | + | +^#^ | += | + | + | - | + | - | - | - |
|  |  |  |  |  |  |  |  |  |  |  |  |  |  |  |  |  |
| Monteny et al.^[19]^ | + | + | + | + | + | + | + | + | += | + | + | + | + | - | - | - |
|  |  |  |  |  |  |  |  |  |  |  |  |  |  |  |  |  |
| Van den Bruel et al.^[4]^ | + | +~ | + | + | + | - | + | +^#^ | += | + | + | - | +’ | - | - | - |
|  |  |  |  |  |  |  |  |  |  |  |  |  |  |  |  |  |

^&^ “bad capillairy refill” as proxy for “poor colour”

~ “somnolent” as proxy for “no response to social cues”

^ “dull” as proxy for “no response to social cues”

^*^ “disturbed consiousness” as proxy for “does not (stay) awake”

^#^ “dyspneu” as proxy for “tachypneu” or “chest indrawing”

= “signs of dehydration” as proxy for “reduced skin turgor”

‘ “meningeal irritation” as proxy for “neckstiffness”

|  |
| --- |

= “General” red traffic lights

|  |
| --- |

= “Disease specific” red traffic lights
