# Supplementary material for: The Predictive Value of the NICE “Red Traffic Lights” in Acutely Ill Children
Source: PLoS One. 2014 Mar 14;9(3):e90847. doi: 10.1371/journal.pone.0090847 (PMC3954615; doi:10.1371/journal.pone.0090847)
Supplement: Table S2 — Missing/not recorded data. (DOC) [file pone.0090847.s002.doc]

| Dataset n cases prevalence % missing/ not recorded red traffic lights | | | | | | | | | | | | | | | | | | |
| --- | --- | --- | --- | --- | --- | --- | --- | --- | --- | --- | --- | --- | --- | --- | --- | --- | --- | --- |
| COLOUR ACTIVITY RESPIRATORY HYDRATION OTHER | | | | | | | | | | | | | | | | | | |
|  |  |  | Colour | No response to social cues | Ill appearance | Does not (stay) awake | Quality of cry | Grunting | Tachypnoea | Chest indrawings | Reduced skin turgor | Age <3m & temp ≥38°C | Non-blanching rash | Bulging fontanelle | Neck stiffness | Status epilepticus | Focal neurologic signs | Focal seizures |
| Bleeker et al.^[23]^ | 595 | High | 13.95% | # | 6.89% | 42.86% | 13.28% | # | 9.41% | # | 7.56% | 0.00% | # | 47.73% | # | # | # | # |
|  |  |  |  |  |  |  |  |  |  |  |  |  |  |  |  |  |  |  |
| Brent et al.^[18]^ | 494 | High | 1.82% | 2.22% | 1.21% | 1.42% | # | 0.00% | 0.00% | 0.00% | 1.21% | 0.00% | 0.00% | 0.00% | 0.00% | 0.00% | 0.00% | 0.00% |
|  |  |  |  |  |  |  |  |  |  |  |  |  |  |  |  |  |  |  |
| Oostenbrink et al. ^[22]^ | 423 | High | 0.00% | 34.75% | 48.94% | 0.00% | 34.28% | # | # | # | 73.52% | 0.00% | 0.00% | 27.90% | 0.00% | # | 35.93% | 35.93% |
|  |  |  |  |  |  |  |  |  |  |  |  |  |  |  |  |  |  |  |
| Roukema et al. ^[20]^ | 1459 | High | 46.54% | # | 50.65% | 66.96% | 0.00% | 0.00% | 51.47% | 46.81% | 0.00% | 0.27% | 0.00% | 0.00% | 49.90% | 0.00% | 0.00% | 0.00% |
|  |  |  |  |  |  |  |  |  |  |  |  |  |  |  |  |  |  |  |
| Thompson et al. ^[21]^ | 434 | High | 0.00% | 0.00% | 0.00% | 0.00% | 4.61% | # | 15.67% | 0.00% | 0.00% | 0.00% | 0.00% | # | 0.00% | # | # | # |
|  |  |  |  |  |  |  |  |  |  |  |  |  |  |  |  |  |  |  |
| Monteny et al.^[19]^ | 487 | Low | 1.23% | 1.03% | 5.54% | 3.70% | 3.70% | 0.82% | 24.64% | 8.42% | 0.62% | 0.00% | 7.00% | 5.75% | 2.46% | # | # | # |
|  |  |  |  |  |  |  |  |  |  |  |  |  |  |  |  |  |  |  |
| Van den Bruel et al.^[4]^ | 2468 | Low | 1.06% | 0.93% | 6.21% | 1.06% | 1.94% | # | 1.10% | 0.89% | 1.69% | 1.18% | 1.60% | # | # | # | # | # |
|  |  |  |  |  |  |  |  |  |  |  |  |  |  |  |  |  |  |  |

**Table S2: Missing/ not recorded data**

^#^ Not recorded

= “General” red traffic lights

|  |
| --- |

|  |
| --- |

= “Disease specific” red traffic lights
